# Supplementary material for: Characterising people who inject drugs, and association with HIV infection: A situation analysis in Kampala City, Uganda
Source: PLOS Glob Public Health. 2025 Feb 26;5(2):e0003370. doi: 10.1371/journal.pgph.0003370 (PMC11864543; doi:10.1371/journal.pgph.0003370)
Supplement: Attachment — Submitted filename: Response to Reviewers Nov 8 24.doc [file pgph.0003370.s003.doc]

**Response to Reviewers**

**PLOS Global Public Health Decision: Revision required [PGPH-D-24-01264] - [EMID:46db00935ebf3db3]**

**Title: Characterising People who inject drugs, and association with HIV infection: A Situation Analysis in Kampala city, Uganda**

Dear Editor,

The following documents have been attached as requested, and below is a narrative of our responses to the detailed comments.

- A rebuttal letter responding to each point raised by the editor and reviewer, labelled 'Response to Reviewers'.
- A marked-up copy of the manuscript highlighting changes made to the original version. labelled 'Revised Manuscript with Track Changes'.
- An unmarked version of the revised paper without tracked changes, labelled 'Manuscript'.
- Figure files saved in .tif submitted separately
- A Complete version of the questionnaire is uploaded file named “**Inclusivity in global research questionnaire**

**Journal Requirements:**

1. Please ensure you have stated the type of consent (Written) in the Methods section of your manuscript text to fully comply with the PLOS ONE policy on reporting research involving human participants. This information is currently provided only in the Human Participants Research Checklist, which will not be published with your manuscript files.

***Response:*** **Thanks for this guidance, on page 5, under the section “Ethical Considerations”, the word written has been added and now the second sentence reads “All the participants were interviewed after providing written informed consent*.”***

2. Please include a complete copy of PLOS’ questionnaire on inclusivity in global research in your revised manuscript. Our policy for research in this area aims to improve transparency in the reporting of research performed outside of researchers’ own country or community. The policy applies to researchers who have travelled to a different country to conduct research, research with Indigenous populations or their lands, and research on cultural artefacts. The questionnaire can also be requested at the journal’s discretion for any other submissions, even if these conditions are not met. Please find more information on the policy and a link to download a blank copy of the questionnaire here: https://journals.plos.org/globalpublichealth/s/best-practices-in-research-reporting. Please upload a completed version of your questionnaire as Supporting Information when you resubmit your manuscript.

**Response: A Complete version of the questionnaire is uploaded file named “Inclusivity in global research questionnaire”**

3. In the online submission form, you indicated that [The datasets analysed during the current study will be shared on reasonable request by the corresponding].

All PLOS journals now require all data underlying the findings described in their manuscript to be freely available to other researchers, either

a. In a public repository,

b. Within the manuscript itself, or

c. Uploaded as supplementary information.

This policy applies to all data except where public deposition would breach compliance with the protocol approved by your research ethics board. If your data cannot be made publicly available for ethical or legal reasons (e.g., public availability would compromise patient privacy), please explain your reasons by return email and your exemption request will be escalated to the editor for approval. Your exemption request will be handled independently and will not hold up the peer review process, but will need to be resolved should your manuscript be accepted for publication. One of the Editorial team will then be in touch if there are any issues.

**Response: We appreciate your insightful comments and confirm that we have submitted data file supporting the findings described in the manuscript as supplementary csv file named “*profiling PWID data set uga 15may24”***

4. Please provide separate figure files in .tif or .eps format.

**Response: The figures in this manuscript are being submitted in separate files .tif format**

**Major comments:**

**1. HIV testing:** There are two places in the manuscript that described the way HIV testing was done (“study measurements” section in the methods and “HIV prevalence” section in discussion), and they describe it in very different ways. This inconsistency needs to be resolved. Importantly, in the discussion section, they indicated that participants who self-reported a recent negative test (past 3 or 6 months? Timeframe is inconsistently written, too) did not undergo HIV rapid testing through the study. This is not a reliable way to estimate HIV prevalence. And indeed, the study found quite low HIV prevalence. With the ambiguity of HIV testing method, I am not sure about the validity of this finding.

**Response: We acknowledge the confusion caused by inconsistencies in describing the establishment of HIV serostatus in the two sections mentioned. In this study, participants who self-reported a positive test were not retested, while those who reported a negative test result were tested according to the national algorithm to confirm HIV status. We have therefore removed the section in the discussion referring to self-reports among individuals with negative results. Instead, we provide a narrative explaining the potential reasons for low HIV positivity within this population, supported by citations from previous studies in Uganda that reported similarly low HIV prevalence among injecting drug users. Please refer to the subsection on HIV prevalence under the discussion section on page 14 of 21, highlighted with track changes.**

2. Frequency of injection drug use, types of drugs injected and non-injection drug use: In “Drug use-related characteristics” section in the results, they report that 50% of their sample last injected drugs more than a month before the interview. This suggests that study participants are mostly very occasional injectors, and is not aligned with conventional characteristics of PWID discussed in the context of HIV/AIDS (i.e., usually people inject drugs on a much more frequent basis). Despite this, the authors do not discuss this point in the discussion section – is it common in Uganda that people inject drugs so infrequently or is it just that the study did not reach those who engage in more intense injection drug use patterns?

**Response:** The omission of injecting frequency among drug users in Uganda was unintentional. We acknowledge that most injectors in Uganda are occasional injectors. Therefore, we have revised the previous statement:

**“The study reveals the existence of risky drug injection and sexual practices in Uganda with the potential to increase the number of new HIV infections. The common risk drug injection practices reported include sharing needles and syringes, sharing mixing equipment, flushing blood, and reusing syringes multiple times”**

to read as …

**“Although most participants were found to be occasional injectors, risky drug injection and sexual practices were common, posing a significant potential for transmitting and increasing the number of new HIV infections. Reported risky injection practices included sharing needles and syringes, sharing mixing equipment, flushing blood, and reusing syringes multiple times”. Refer to section on “Risk factors for HIV infection” page 15**

**Importantly the observation of less frequent injecting beavoiur may explain the low uptake of the needle syringe program(NSP) currently being introduced into the country**

Also, they do not report any types of drugs injected or non-injection drug use patterns. Without such information, they cannot claim that they are providing “a comprehensive insight” into the characteristics of PWID in Kampala. The low frequency of injection drug use may mean that those participants were engaged more in non-injection drug use.

**Response: We acknowledge that the frequency of drug injecting in Uganda is low, a finding supported by previous studies cited in the discussion section(refer to page 14). Additionally, we have included Figure2 and a narrative detailing the types of drugs used and the frequency of use; refer to page 7, under the section “Drug use-related characteristics”.**

3. Multivariable regression modeling procedure (statistical analysis section in the methods) is unclear. They stated that they included “variables that had p values <0.1 or known association with HIV serostatus, were selected for inclusion.” Which variables are the known association with HIV serostatus? Were variables that were not hypothesized to be associated with HIV serostatus but had p values <0.1 still included in the multivariable model? They should not.

**Response: This guidance is well noted, in the analysis, we included all variables with P-values<0.1. In the “Statistical Analysis” section describing variables included in the multivariate analysis, the statement “or known association with HIV serostatus” has been deleted, refer to page5.**

4. In “Risk factors for HIV injection” in the discussion, the authors stated that: “The common risk drug injection practices reported include sharing needles and syringes, sharing mixing equipment, flushing blood, and reusing syringes multiple times.” But the prevalence of these behaviours was less than 10%. I don’t think we call it “common” behaviour. Again, this raises a question about who the study sample was – weren’t they mostly drug smokers, not injectors?

**Response: Thanks for this comment, we do agree that these risk factors were not very common especially compared to what has been reported elsewhere, and probably due to the less frequent injecting practices reported in our study. The statement stating that “The common risk drug injection practices reported include sharing needles and syringes, sharing mixing equipment, flushing blood, and reusing syringes multiple times” has been reworded to read “The reported risk drug injection practices, although infrequent, include sharing needles and syringes, sharing mixing equipment, flushing blood, and reusing syringes multiple times” see page15**

5. “HIV Risk behavior” and “Drug use and sexual-related HIV risk factors” sections in the results include some interpretation of the results (e.g., “…was a significant concern”). Such interpretation should be removed from the results section and placed in the discussion section.

**Response: We acknowledge that the results section should focus solely on presenting data, without including any interpretations. On page 9 under the subsection “HIV Risk behavior”, We have therefore reworded the statement “Sharing of injecting equipment was a significant concern, with 33 participants (9.3%) admitted to using a shared needle or syringe, and a similar proportion acknowledging the sharing of other injecting materials”, to read “Thirty-three participants (9.3%) reported using a shared needle or syringe, with a similar proportion acknowledging the sharing of other injecting materials.”**

**In the section “Drug use and sexual-related HIV risk factors”, the first sentence has been revised to remove interpretations. It now reads “Regarding sexual behaviors, 189(53.4%) participants reported engaging in sexual encounters with non-regular partners within the last three months, of whom 19.8% reported that they knew the HIV status of their non-regular partners.” Refer to page 9.**

6. “Risk factors for HIV injection” in the discussion section includes a discussion of non-nationals, and the authors stated that: “The risk of being HIV positive was found to be five times higher among non-nationals compared to Ugandans.” As the regression results did not show a statistically significant association and the 95% CI is very wide, this statement as well as the related discussion should be removed.

**Response: We agree with this recommendation. Under the section on Risk factors for HIV infection, under the discussion section, we have edited this statement stating that “The risk of being HIV positive was found to be five times higher among non-nationals compared to Ugandans.” , that reflects the observation of having high proportions of non-Ugandan opioid injectors who were found HIV positive but with no significant association. The narrative now reads “who were mostly refugees of recent immigration status. High proportions of non-Ugandans were found HIV positive but with no significant association in multivariate analysis.” Please refer to page 15**

Minor comments

7. Introduction, 1st para: “The rising burden of drug use including injecting, continues to harm the economic, health, and social sectors(1).” This sentence is misleading and should be revised. It is not the drug use per se that harms the society, but the way drug use is regulated and viewed in the society.

**Response: We have noted this recommendation and made the changes based on the cited reference. In the introduction paragraph 1, the statement “The rising burden of drug use including injecting, continues to harm the economic, health, and social sectors” has been edited to: “ The rising burden of *illicit drug use* including injecting, continues to harm the economic, health, and social sectors” (see page 3)**

8. “Study population, sampling and sample size” and “Study procedure” sections in the methods can be merged. The two sections include descriptions of how the researchers recruited participants.

**Response: We appreciate the observation that the two sections appear similar. We also note that we had named the section on data collection as “study procedure”. In this revised paper, under methods page 4, we have replaced the subtitle “study procedure” with “data collection”**

9. Abstract, methods: The authors stated that they used “a semi-structured questionnaire”; however, it should be a structured questionnaire. A semi-structured questionnaire is usually used for qualitative interviews.

**Response: We agree with this recommendation. Throughout the manuscript, we have replaced the word “semi-structured questionnaire” with the word “structured questionnaire “**

10. “Mental health” section in the discussion: The two sentences are conflicting as ADHD appears in both sentences. This should be corrected. “Depression, Anxiety, and Attention Deficit Hyperactivity Disorder were the most prevalent co-morbidities. Schizophrenia, post-traumatic stress disorder (PTSD), and attention deficit hyperactivity disorder (ADHD) were less common in this population.”

**Response: We highly appreciate this observation and recommendation. In this study, we found that ADHD occurred in 33(6.6%) of the participants which is considered to be a common occurrence therefore we delete the second part that refers to ADHD as less common. Henceforth on page 12, the second mention of the word “Attention Deficit Hyperactivity Disorder” has been deleted**.

11. Overall, English writing and typos should be checked and corrected.

**Response: This is noted. We have re-read the manuscript to correct all the typos and grammatical errors (see attached manuscript)**
