# Supplementary material for: Characterising people who inject drugs, and association with HIV infection: A situation analysis in Kampala City, Uganda
Source: PLOS Glob Public Health. 2025 Feb 26;5(2):e0003370. doi: 10.1371/journal.pgph.0003370 (PMC11864543; doi:10.1371/journal.pgph.0003370)
Supplement: Attachment — Submitted filename: Response to reviewers comments Jan 25.docx [file pgph.0003370.s004.docx]

**Response to Reviewers**

**PLOS Global Public Health Decision: Revision required [PGPH-D-24-01264] - [EMID:46db00935ebf3db3]**

**Characterising People who inject drugs, and association with HIV infection: A Situation Analysis in Kampala city, Uganda**

Dear Editor,

Thanks for comprehensive review and feedback to improve our manuscript. With your guidance, we have made the revisions and detailed response to the your comments and corrections are detailed below. In addition, we have attached the following items:

- A rebuttal letter that responds to each point raised by the editor and reviewer(s). You should upload this letter as a separate file labelled 'Response to Reviewers'.
- A marked-up copy of your manuscript that highlights changes made to the original version. You should upload this as a separate file labelled 'Revised Manuscript with Track Changes'.
- An unmarked version of your revised paper without tracked changes. You should upload this as a separate file labelled 'Manuscript', among other documents.

**Responses**

**Reviewer #1: The authors addressed most of my previous comments. I have a few minor comments.**
1) The sample size seems to have been reduced from 499 to 354. However, there is no explanation for it. Why has it been reduced?

**Response: We acknowledge the observation on the change in sample size.**

**Initially, we had 2 sample sizes reported. In Tables 1 and 2 we had a sample size of 499 that included 145 non-injecting drug users; in Tables 3 and 4 we had a sample size of 354. To align with the title (only people who inject drugs), and avoid confusion, we decided to use a sample size of 354, that includes only injecting drug users, hence the reason for the change in sample size.**

2) Abstract, Conclusion, and the Conclusion section in the manuscript: "Our study provides a comprehensive insight into..." I still think that the term "comprehensive" is a stretch. I suggest that the authors tone it down.

**Response: Thanks for this comment, we do agree with the suggested change in the conclusion. In the abstract and conclusion section, the word “a comprehensive” has been deleted and replaced with “valuable” and the sentence now reads “Our study provides valuable insight into the socio-demographic, mental, physical health, and HIV risk behaviour of PWID in Kampala, Uganda.”(see pages 2 and 15)**

3) Introduction: "the rising burden of illicit drug use including injecting, continues to harm the economic, health, and social sectors." Again, I would argue that the current ways in which (illicit) drug use is regulated are causing the harm, not the (illicit) drug use per se. I defer to the Editor on this point.

**Response: This comment is well noted. We agree that although drug use is a problem, the challenges associated with its regulation cause more harm. We have henceforth revised the sentence to include the argument on challenges associated with regulating drug use. The revised sentence now reads “The rising burden of illicit drug use including injecting *and challenges related to its regulation*, continues to harm the economic, health, and social sectors (see last sentence first paragraph under introduction section page 3)**

4) Results, "HIV risk behaviour" and "Drug use and sexual-related HIV risk factors" sections: These sections still contain some interpretation of the results. I defer to the Editor on this point.

**Response: We acknowledge that the results section should focus solely on presenting data, without including any interpretations. The Results sections on "HIV risk behaviour" and "Drug use and sexual-related HIV risk factors" have both been revised and all text related to interpretation of results have been deleted. (see page 9 subsections “"HIV risk behaviour" and "Drug use and sexual-related HIV risk factors” under track changes**

5) Discussion, HIV prevalence: It is reported as 4.38%. However the results section reports it as 3.7%. The discrepancy needs to be resolved.

**Response: This observation is well noted. When we computed the HIV prevalence based on the sample size 354, the resulting prevalence was 3.7% which we erroneously forgot to update in the discussion section. We have since corrected and replaced the 4.38% in the discussion section with the value of 3.7% (see first sentence subsection “HIV Prevalence” under discussion section, subsection on** **“HIV prevalence” on page 13)**

Reviewer #2: Thank you for this revised version. I would encourage you to reflect further on the comments and ensure that the abstract and paper - throughout - are coherent. As an example, you note in the limitations, there were some issues with gaining access to the population for this study, so it is not correct to claim this provides comprehensive insights.

**Response: This guidance is well noted, we agree that studies among populations such as injection drug users engaging in behaviours considered illegal in certain setting may not be able to access the populations exhaustively and therefore, “comprehensive” insights may be a strong conclusion. We have therefore replaced the word “comprehensive” with “valuable” in both the abstract and main manuscript conclusion subsections(see pages 2 and 15)**

I would encourage you to be very careful with the use of language - `HIV positives' is not acceptable since it defines a person by an infection. Please follow the People First Charter language. Likewise, the term `developing countries' was replaced many years ago by other terms.

**Response: Thanks for this very important observation and advise. We have replaced the word “HIV positives” with persons living with HIV’ which is a person-centered, non-stigmatizing, and respectful terminology (see page 9, under subsection on “HIV positivity among people who inject drugs” ). Likewise, under discussion paragraph 2 on page 12, we have replaced the word “developing countries” with a more appropriate word “Low- and Middle-Income Countries”**

Page 4 - rather than using the word `hotspots' describe the locations. What types of place did you meet people?
**Response: This advice is well noted. In the entire manuscript writeup, we have replaced the word “hotspot” with “venue”(including bars, lodges, street corners and ghettos), see track changes in the manuscript**

Page 5 and elsewhere - do you mean “flash blood syringes” or “flashblood”? And I suggest you explain what this is - some readers may not know.

**Response: Thanks so much for the clarification and guidance. We have revised the manuscript to replace the word “flash blood syringes” to “flash blood in the narrative (see track changes )**

Page 5 - was clearance gained from UNCST and CDC in addition to the Makerere REC?
**Response: In this study, we sought clearance from school of Public health (protocol number SPH-2021-166) and administrative clearance from Butabika National referral hospital(see attached letters from IRB)**

Page 6 - Roman Catholic - consider use of capital letters for religious denominations/faiths since you have now used that for Anglican.

**Response: We acknowledge this guidance. We have revised the narrative to use capital letters for first letter of the names of the religious denominations (see track changes in the revised manuscript)**

The paper requires very careful language editing. There are inconsistences - for example, data were/data was (use date were throughout). I suggest you employ an English language editor to help you.

**This guidance is well appreciated. We have revised the entire write up to replace the words “data was” with “data were”, in line with guidance provided.**
